# Orbital Roof Blowout Fracture With an Intact Orbital Rim: A Case Report

**Published:** 2020-11-09

**Authors:** Kun Hwang, Se Yang Oh

**Affiliations:** ^a^Department of Plastic Surgery, Inha University School of Medicine, Incheon, Korea; ^b^Department of Neurosurgery, Inha University Hospital, Incheon, Korea

**Keywords:** orbital fractures, orbit, tomography, x-ray computed, craniotomy

## Abstract

**Background:** Fractures of the orbital roof not associated with fractures of the orbital rim are unusual. We describe the case of a blowout fracture of the orbital roof with an intact orbital rim, which was found after craniotomy for removal of epidural hematoma.

**Case:** A 64-year-old man was referred to our emergency department from a local hospital. He fell down from a 3-m stepladder while pruning branches of a tree. Brain computed tomographic scan revealed acute epidural hematoma in both frontal convexities, and he underwent craniotomy at the local hospital. On follow-up brain computed tomography, an orbital roof fracture with a displaced bony fragment and hemorrhage was noticed in the left superior extraconal space. Thereafter, he was transferred to our department. Upon examination, movement of the extraocular muscles was normal. He did not complain of diplopia or decreased sensation of the face. He also did not have nasal stuffiness. Exophthalmometry revealed the same findings for both eyes (18 mm/18 mm). Facial computed tomographic scan before the second operation revealed a displaced orbital roof fracture segment. Under general anesthesia, craniotomy was performed and the epidural hematoma was evacuated. The displaced bony fragment was removed from the left anterior cranial fossa, and the anterior skull base was reconstructed with a titanium mesh plate.

**Conclusion:** Through this case of blowout fracture of the orbital roof with an intact orbital rim, found after craniotomy, we should be aware of the possibility that an orbital roof fracture can be missed on conventional brain computed tomography.

Most blowout fractures involve the orbital floor. Less often, the medial orbital wall is fractured, either alone or in conjunction with the floor.^1^ Upward displacement fractures do not occur in isolation but may accompany extensive craniofacial fractures and usually involve the orbital rim.^2^

If the roof of the orbit is thin and direct compressive or buckling forces impact the orbit, the fracture can involve the upper roof. Fractures of the orbital roof not associated with fractures of the orbital rim are unusual.^3^

We describe the case of a blowout fracture of the orbital roof with an intact orbital rim, which was found after craniotomy for removal of epidural hematoma.

This study was approved by the institutional review board (IRB) of Inha University Hospital (IRB No. 2020-08-005-000).

## CASE

**Figure 1:**
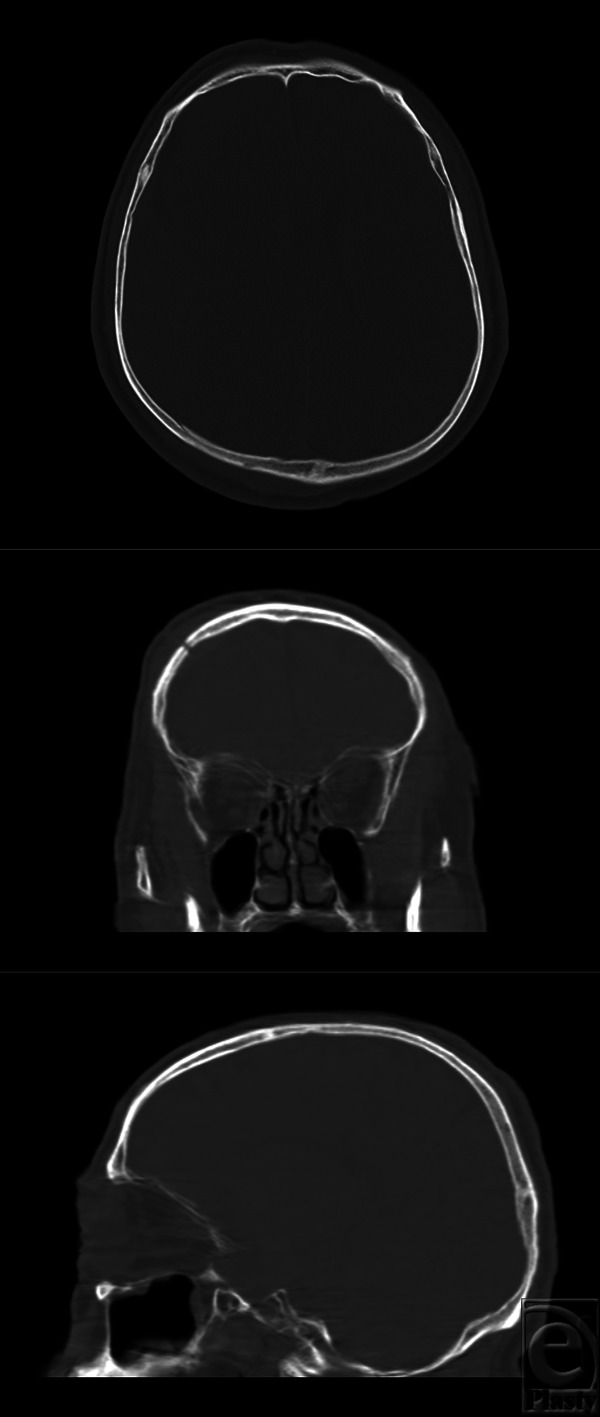
Initial brain computed tomography at the local hospital. Upper: axial view. Middle: coronal view. Lower: sagittal view. No definite fracture is seen on the left orbital roof. Parietal bone fractures are seen in the sagittal view.

**Figure 2:**
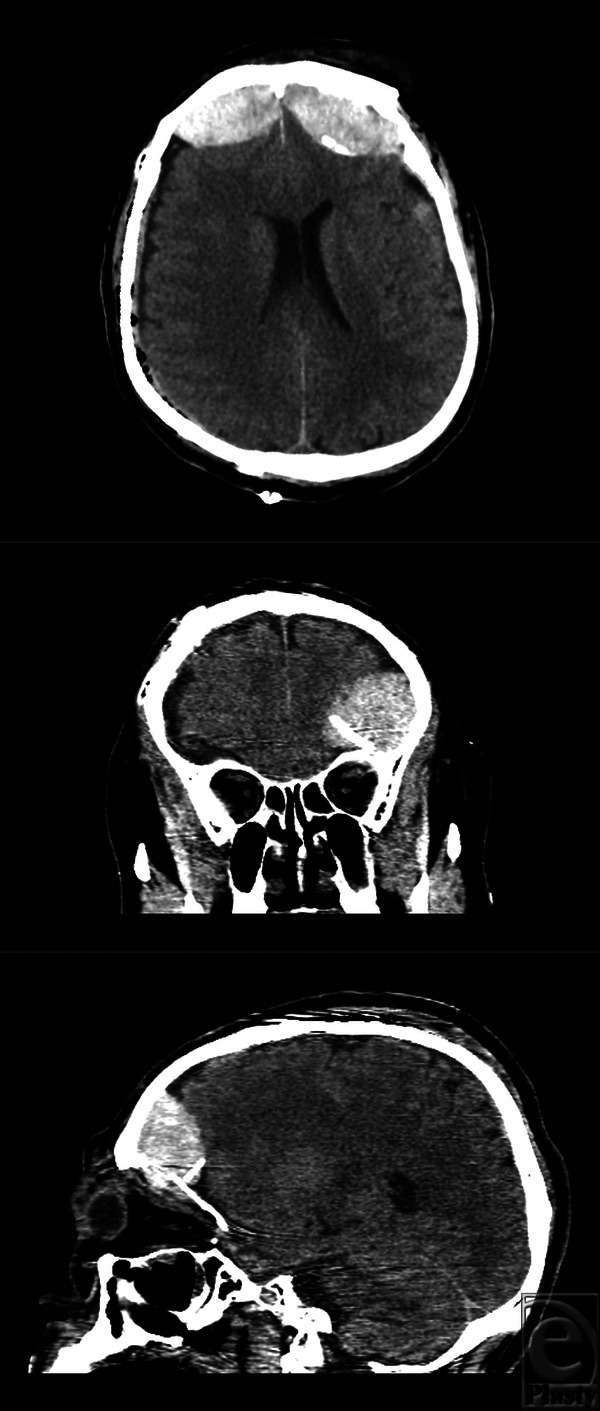
Brain computed tomography after the first operation at the local hospital. Upper: axial view. Middle: coronal view. Lower: sagittal view. Epidural hematomas are seen on both anterior frontal convexities. A displaced orbital roof fracture segment is seen.

A 64-year-old man was referred to our emergency department from a local hospital. He fell down from a 3-m stepladder while pruning branches of a tree. Brain computed tomographic (CT) scan revealed acute epidural hematoma in both frontal convexities (Fig 1), and he underwent craniotomy at the local hospital. On follow-up brain CT, an orbital roof fracture with a displaced bony fragment and hemorrhage was noticed in the left superior extraconal space. Thereafter, he was transferred to our department (Fig 2).

**Figure 3:**
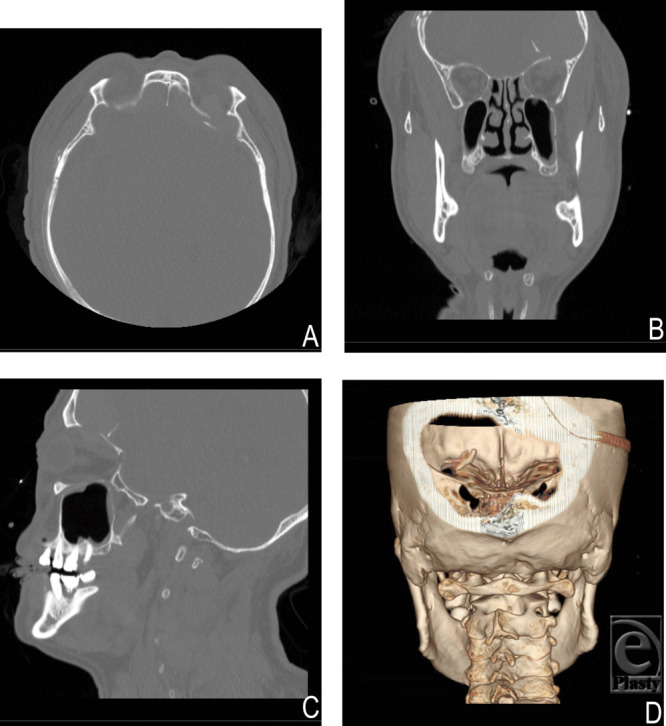
Facial computed tomography before the second operation: (*a*) axial view; (*b*) coronal view; (*c*) sagittal view; and (*d*) 3-dimensional reconstructive view. A displaced orbital roof fracture segment is seen.

Upon examination, movement of the extraocular muscles was normal. He did not complain of diplopia or decreased sensation of the face. He also did not have nasal stuffiness. Exophthalmometry revealed the same findings for both eyes (18 mm/18 mm). Facial CT scan before the second operation revealed a displaced orbital roof fracture segment (Fig 3).

**Figure 4:**
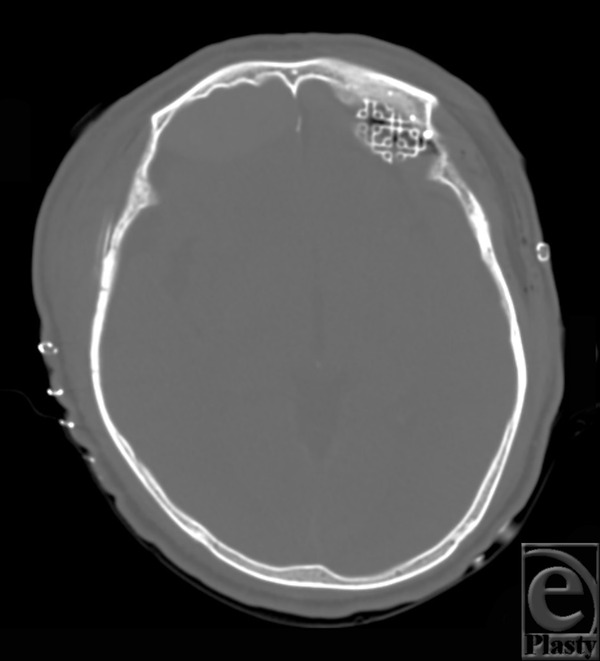
Brain computed tomography after the second operation, axial view. A displaced orbital roof fracture segment has been removed, and the defect of the orbital floor was reconstructed with a titanium mesh plate.

Under general anesthesia, craniotomy was performed and the epidural hematoma was evacuated. The displaced bony fragment was removed from the left anterior cranial fossa, and the anterior skull base was reconstructed with a titanium mesh plate (Optimus neuroplate, 3D Mesh 50×50 mm; Osteonic, Seoul, Korea) (Fig 4).

## DISCUSSION

The orbital globe can prolapse into the anterior cranial fossa through fractures of the superior orbit roof.^4^ The displacement of the supraorbital rim and the orbital roof is generally downward and posterior, producing an anterior and inferior displacement of the globe and orbital soft tissue. Ptosis of the eyelid is present, depending on the degree of displacement, and the contusion of the extraocular muscles first includes the levator.^5^ In the present case, the eyeball was not trapped in the fractured orbital roof and the patient did not have diplopia or restriction of extraocular muscle movement.

When surgeons notice the displacement of the orbital globe comparing with the contralateral side or blepharoptosis after head and facial trauma, further workup for the orbital roof fracture is required.^5^

An orbital roof fracture without an orbital rim fracture can be blown downward by distant skull fractures. These results from a sudden increase in intracranial pressure transmitted through the anterior fossa and decompressed by a fracture of the orbital roof.^6^ In the present case, the skull fractures were located in the parietal bone.

Isolated fractures of the orbital roof occur rarely and are usually found when a thin anterior skull base is present. This thinness makes reduction of the fragments difficult and unstable. For these types of fractures, there exist several reconstruction options, including alloplastic materials and titanium mesh plates.^3^ For our patient, we chose to use a titanium mesh plate.

Orbital roof fractures are usually picked up when the patients are admitted and undergo radiological examinations unless life-threatening neurological and neurosurgical complications are present. However, late presentations may appear approximately 2 years after trauma.^7^ However, early diagnosis and treatment are very important since complications such as irreversible damage to the optic nerve and ascending infections favored by a cerebrospinal fluid fistula can occur.^3^ In the present case, a blowout fracture of the orbital roof was found after craniotomy for removal of the epidural hematoma at a local hospital.

Surgical repair of the orbital roof fracture is usually combined with neurosurgical procedures and ideally recommended within 2 weeks when symptomatic diplopia is present along with a positive forced duction test and evidence of orbital soft-tissue entrapment on the CT scan, or in patients with large fractures that may cause latent enophthalmos or hypo-ophthalmos, as Burnstine^8^ insisted for orbital floor fracture.^9^ However, repair should be delayed until the traumatic brain injury has subsided. After the anterior cranial fossa and the frontal region are exposed and the brain and dural repairs are completed, the orbital roof defect should be repaired by a thin bone graft placed external (within the cranial cavity and not the orbit) to the intact rim of bone in the orbital roof.

Isolated roof fractures may be accompanied by serious ocular complications, such as optic nerve contusion and visual injury loss or superior orbital fissure syndrome, orbital apex syndrome, and rarely cavernous sinus fistulae. Significant fractures of the orbital roof extend posteriorly to involve the superior orbital fissure and optic foramen. Involvement of the superior orbital fissure produces the superior orbital fissure syndrome.^10^^,^^11^ This involves the following structures: the 2 divisions of the cranial nerve III, superior and inferior, producing paralysis of the levator, superior rectus, inferior rectus, and inferior oblique muscles. Cranial nerve IV causes paralysis of the superior oblique muscle, and cranial nerve VI produces paralysis of the lateral rectus muscle. The ophthalmic division of the trigeminal nerve (V) causes anesthesia in the brow, medial portion of the upper lid, medial upper nose, and ipsilateral forehead. If involvement of both the optic nerve and the superior orbital fissure occurs, this symptom complex is called the orbital apex syndrome.^12^ The intracavernous segment of the internal carotid artery may be ruptured when a sudden acceleration-deceleration force is applied^13^ and also be lacerated by bony spicules from fractures of the anterior clinoid processes and the orbital roof.^14^

Through this case of blowout fracture of the orbital roof with an intact orbital rim, found after craniotomy, we should be aware of the possibility that an orbital roof fracture can be missed on conventional brain CT.
